# Impact of injectable vitamin B12 among malnourished Indian children

**DOI:** 10.6026/973206300221491

**Published:** 2026-03-31

**Authors:** Shubham Saniyar, Asmita Chakraborty, Pushkar Singh Parihar, Meena Patel, Jeetendra Singh

**Affiliations:** 1 Department of Pediatrics, Shyam Shah Medical College Rewa, Madhya Pradesh, India

**Keywords:** Severe acute malnutrition (SAM), vitamin B12 deficiency, neurodevelopmental delay, injectable vitamin B12

## Abstract

Severe Acute Malnutrition (SAM) is often associated with micronutrient deficiencies, including vitamin B12, which is crucial for hematological function and neurodevelopment. Vitamin B12 deficiency in early childhood can result in clinical illness, developmental delay and regression of developmental milestones. Therefore, it is of interest to assess the impact of injectable vitamin B12 supplementation on clinical and neurodevelopmental outcomes in children aged 6-30 months with SAM and vitamin B12 deficiency. The study used clinical, neurological and neurodevelopmental assessments before and after three months of supplementation. This study advances our understanding of the role of vitamin B12 supplementation in improving both clinical and neurodevelopmental outcomes in children with SAM and deficiency.

## Background:

Malnutrition continues to be a major public health challenge in low- and middle-income countries, with India bearing a considerable burden of childhood under nutrition and micronutrient deficiencies. Severe Acute Malnutrition (SAM) is associated with increased risk of morbidity, mortality and adverse neuro-developmental outcomes in children under five years of age [1]. Vitamin B12 is a crucial micronutrient involved in DNA synthesis, red blood cell production and normal neurological functioning. It plays an essential role in myelination, neurotransmitter production and neuronal maturation during early childhood, a critical phase for brain development [2]. Deficiency of vitamin B12 in infancy and early childhood has been linked to hypotonia, delayed development, regression of milestones, involuntary movements, anemia and impaired cognitive function [3]. Children with SAM are at particularly high risk of vitamin B12 deficiency due to inadequate dietary intake, malabsorption, frequent infections and increased metabolic requirements [4]. Several studies have reported a high prevalence of vitamin B12 deficiency among malnourished children, especially in populations with predominantly vegetarian dietary practices [5]. Neuro-developmental impairment associated with vitamin B12 deficiency may manifest as delayed acquisition of motor and mental milestones, loss of previously achieved skills, irritability, hypotonia and characteristic neuroimaging abnormalities such as delayed myelination and cerebral atrophy [61]. Early recognition and treatment of vitamin B12 deficiency are crucial, as prolonged deficiency can result in permanent neurological damage [7]. Although parenteral vitamin B12 therapy is known to rapidly correct hematological and biochemical abnormalities, there is limited prospective evidence evaluating its effect on neuro-developmental outcomes in children with SAM [8]. In view of the limited Indian data examining both clinical and neuro-developmental outcomes follows vitamin B12 supplementation. Therefore, it is of interest to evaluate the impact of Injectable vitamin B12 therapy in children with severe acute malnutrition and vitamin B12 deficiency.

## Methodology:

This was a prospective cohort study conducted from July 2023 to June 2024 in the SMTU Ward of the Department of Pediatrics at Gandhi Memorial Hospital, associated with Shyam Shah Medical College, Rewa (MP), involving children aged 6-30 months admitted with severe acute malnutrition and vitamin B12 deficiency. The inclusion criteria were children aged 6-30 months with Severe Acute Malnutrition, defined by any one of the following: weight-for-length/height < -3 SD, mid-upper arm circumference (MUAC) < 11.5 cm, symmetrical bilateral pedal edema of nutritional origin, or serum vitamin B12 level < 200 pg/dL. Exclusion criteria included children with neurodevelopmental delay due to causes other than vitamin B12 deficiency, such as sequelae of hypoxic-ischemic encephalopathy (HIE), neuronal migration disorders, congenital brain malformations, or structural brain abnormalities detected on neuroimaging not attributable to vitamin B12 deficiency. All enrolled children received injectable vitamin B12 (1000 µg intramuscular) administered on alternate days during the first week, followed by weekly injections for two months and then monthly thereafter. Outcome assessment involved clinical outcomes such as alertness, hypotonia, irritability, involuntary movements, hyperpigmentation of knuckles and systemic symptoms, along with neurodevelopmental outcomes like developmental delay and regression assessed using the Developmental Assessment Scale for Indian Infants (DASII). Neuroimaging with MRI brain was conducted in children with abnormal neurodevelopmental assessments and follow-up was carried out at 3 months. Data were entered and managed using Microsoft Excel and statistical analysis was performed using appropriate tests, with continuous variables expressed as mean ± standard deviation and categorical variables as frequencies and percentages. A p-value < 0.05 was considered statistically significant.

## Results:

Table 1 (see PDF) presents the distribution of subjects based on various symptoms across different visits. At Visit 1, 74.9% were alert and 25.1% were lethargic, while at Visit 2, 98.4% were alert. Similarly, 86.9% had normal temperature at Visit 1, with 13.1% showing fever and 99% had normal temperature at Visit 2. The table further highlights the prevalence of pallor (99%), dehydration (24.6% at Visit 1), oral cavity changes, eye symptoms, blood pressure abnormalities and tachypnoea, with all symptoms showing significant improvements at Visit 2 (p < 0.001). Figure 1 (see PDF) illustrates the distribution of subjects by neuroimaging results at the time of admission, while Figure 2 (see PDF) depicts the distribution of subjects with developmental delay and regression. Table 2 (see PDF) compares the Developmental Quotients (DMoQ and DMeQ) at admission and follow-up for the overall study group. The mean DMoQ increased from 68.48 at admission to 81.73 at follow-up (p < 0.01) and the mean DMeQ rose from 65.58 to 75.38 (p < 0.01). Table 3 (see PDF) focuses on subjects with abnormal neurodevelopmental assessments at admission. The DMoQ and DMeQ showed substantial improvement from admission to follow-up, with p values both less than 0.01, indicating significant recovery in these subjects. Table 4 (see PDF) details the developmental quotients of subjects with developmental delay and regression, categorized by isolated and combined DMoQ and DMeQ. A majority of patients (81%) had combined DMoQ and DMeQ < 70% at admission, with some improvement noted at the follow-up visit. Table 5 (see PDF) provides data on subjects with developmental regression, again showing improvement in both DMoQ and DMeQ at follow-up. The mean DMoQ increased from 59.73 to 67.37, while the DMeQ shifted from 65.15 to 64.25, reflecting some improvement, though the majority of subjects remained below the 70% threshold at follow-up. Table 6 (see PDF)examines subjects less than 15 months of age. The DMoQ and DMeQ scores at admission were 67.47 and 65.28, respectively, improving significantly by follow-up, with mean scores rising to 81.41 and 75.40. Table 7 (see PDF) shows similar findings for subjects over 15 months, with the DMoQ and DMeQ scores at admission being 71.24 and 66.41, respectively. These scores increased to 82.63 and 75.31 at follow-up, indicating positive developmental progress. These results underscore the improvements in both motor and mental developmental quotients following treatment, emphasizing the positive impact of the intervention across different age groups and symptom categories. This table shows significant improvement in both motor and mental developmental quotients in children older than 15 months following vitamin B12 supplementation, though the magnitude of improvement was relatively less compared to younger children (Table 7 - see PDF).

## Discussion: 

The present prospective cohort study demonstrates that injectable vitamin B12 supplementation results in significant improvement in clinical condition, neurological manifestations and neuro-developmental outcomes among children with severe acute malnutrition and vitamin B12 deficiency. These findings highlight the essential role of vitamin B12 in early growth and brain development. A notable improvement was observed in systemic clinical features such as lethargy, hypotonia, irritability, dehydration and abnormal vital parameters following vitamin B12 therapy. These changes are likely related to restoration of normal cellular metabolism and improvement in neurological function after correction of cobalamin deficiency, as reported in earlier studies [9]. A considerable proportion of children exhibited abnormal neuroimaging findings at admission, including delayed myelination and cortical atrophy. Similar findings have been documented in vitamin B12 deficient infants and are attributed to impaired myelin formation and neuronal injury [10]. These abnormalities account for the high frequency of neuro-developmental delay and regression observed in the study population.

The significant reduction in developmental delay and regression after supplementation suggests that neurological dysfunction due to vitamin B12 deficiency is potentially reversible when treated promptly. Improvement in developmental regression, in particular, underscores the benefit of early intervention [11]. However, existing literature indicates that prolonged deficiency may lead to residual deficits, emphasizing the importance of early diagnosis and treatment. Motor developmental recovery was greater than mental developmental recovery in the present study. This pattern has been observed previously and may be explained by faster recovery of motor pathways compared to higher cortical functions involved in cognition [12]. Persistently lower mental developmental scores in some children indicate the need for longer follow-up and additional developmental support. Most children presented with combined motor and mental developmental deficits at admission, reflecting the widespread impact of vitamin B12 deficiency on neurodevelopment. Although a significant reduction in combined deficits was observed following treatment, residual deficits in a subset of children may be attributed to the combined effects of malnutrition, micronutrient deficiencies and recurrent infections [13]. Children younger than 15 months showed better neuro-developmental recovery compared to older children, supporting the concept of a critical period for brain development during early infancy when nutritional interventions are most effective [14]. Overall, the findings emphasize the need for routine screening and timely treatment of vitamin B12 deficiency in children with severe acute malnutrition. Incorporating vitamin B12 assessment and supplementation into standard SAM management protocols may help reduce long-term neuro-developmental morbidity.

## Conclusion:

Injectable vitamin B12 supplementation leads to significant improvement in clinical status, neurological features and neuro-developmental outcomes in children with severe acute malnutrition and vitamin B12 deficiency. Early identification and timely intervention are essential to prevent long-term neuro-developmental impairment. Routine screening and appropriate vitamin B12 supplementation should be considered an integral part of the management of children with severe acute malnutrition.
